# Supplementary material for: The validity of small-sided games in predicting 11-vs-11 soccer game performance
Source: PLoS One. 2020 Sep 21;15(9):e0239448. doi: 10.1371/journal.pone.0239448 (PMC7505454; doi:10.1371/journal.pone.0239448)
Supplement: S1 Table — (DOCX) [file pone.0239448.s001.docx]

| Indicator | Outcome | Definition |
| --- | --- | --- |
| Pass forward | Successful – Unsuccessful | A situation in which the attacker attempts to play the ball to a teammate in the forward (i.e., opponent’s goal) direction, by means of his foot/leg/head/torso/sliding. A pass is deemed successful if it reaches the intended teammate and is not touched by a defender. If the ball *is* touched by an opponent, the passing player will only be awarded a successful pass when it is clear that the pass would have also reached the intended teammate without the deflection. It is deemed unsuccessful if it does not reach the intended teammate, or does reach an unintended teammate but was touched/changed direction by an opponent. |
| Dribble | Successful – Unsuccessful | A contest between two or more players in which the attacker attempts to drive by a defender. It is deemed successful if the attacker drives by the defender and maintains possession of the ball. It is deemed unsuccessful if the attacker loses possession of the ball (e.g., often through a successful tackle by the defender). No dribble is awarded if the attacker dribbles in ‘open space’ and does not attempt to drive by a defender. |
| Take on | Successful – Unsuccessful | A contest between two or more players in which the attacker is challenged by the defender, often through physical contact, and aims to maintain control/possession of the ball and/or create space by actions that are not dribbles (e.g., a feint or ‘trick’). It is deemed successful when the attacker maintains control of the ball or creates space to successfully pass to a teammate. It is deemed unsuccessful when the attacker loses possession of the ball. |
| Offensive aerial duel | Successful – Unsuccessful | A contest in the air between two players or more where the attacker (i.e., the player whose team was in possession) attempts to maintain control of the ball, either through passing to a teammate (e.g., by means of a header) or a successful touch. The attempt is deemed successful when the attacker or his teammate maintain possession. It is deemed unsuccessful when he loses possession. |
| Key pass | Counted when it occurs | The final pass that leads to the recipient of the ball having a successful shot attempt without scoring (i.e., a shot on target) |
| Assist | Counted when it occurs | The final pass that leads to the recipient of the ball scoring a goal |
| Shot on target | Counted when it occurs | A scoring attempt that goes into the net (i.e. a goal) or an attempt that clearly would have gone into the net, but was saved by the goalkeeper or a player who is the last line of defense |
| Tackle | Successful – Unsuccessful | A contest between two or more players in which the defender attempts to gain ball possession of an opposing player who is in possession, often through physical contact (e.g., a sliding). The tackle is deemed successful when he successfully takes the ball away from the opposing player, when his teammate gains possession, or when the ball goes out of play and is ‘safe’. It is deemed unsuccessful when he does not gain possession or makes a foul. |
| Staying in front | Successful – Unsuccessful | A contest between two or more players in which the defender attempts to stay in front of an opposing player, often without physical contact, in order to prevent a dangerous offensive (e.g., goal scoring) opportunity. The opposing player attempts to drive by the player, either with a dribble or a running action before receiving a (key) pass. The in front attempt is deemed successful when the opposing player is not able to pass the defending player, and/or is forced to pass the ball to a team mate in a backward or sideways direction. It is deemed unsuccessful when the defender is not able to stay in front of the opposing player. |
| Defensive aerial duel | Successful – Unsuccessful | A contest in the air between two players or more where the defender (i.e., the player whose team was not in possession) attempts to gain control of the ball, either through passing to a teammate (e.g., by means of a header) or a successful touch. The attempt is deemed successful when the defender or his teammate gain possession, or when the ball goes out of play and is ‘safe’. It is deemed unsuccessful when he does not gain possession or makes a foul. |
| Interception | Counted when it occurs | A situation in which the defender ‘reads’ the pass of the opposing player and moves into the line of the intended the pass, thereby intercepting the pass. It is deemed successful when the defender gains possession, or when the ball goes out of play and is ‘safe’. No interception is awarded if the defender accidently receives the ball from the opposing player (e.g., when the defender did not read the pass line, such as picking up a clearance). |
| Applying pressure | Counted when it occurs | A situation in which the defender puts pressure on an opposing player who has ball possession. It is successful when the player in possession loses the ball, often through an unsuccessful pass attempt. A successful pressure attempt can be followed by a tackle, when the defender attempts to conquer the ball through physical contact. |
