# Supplementary material for: The validity of small-sided games in predicting 11-vs-11 soccer game performance
Source: PLoS One. 2020 Sep 21;15(9):e0239448. doi: 10.1371/journal.pone.0239448 (PMC7505454; doi:10.1371/journal.pone.0239448)
Supplement: S2 Table — Coeff. = Estimated Regression Coefficient; SD = Standard Deviation; SE = Estimated Standard Error; The reference group for the factor ‘Team’ is the Under 15 (U15) age category. (DOCX) [file pone.0239448.s002.docx]

| **S2 Table. Multilevel logistic regression analyses for the performance indicators with a successful – unsuccessful outcome in 7-vs-7 and 11-vs-11 games.** | | | | | | | | | | | | | |
| --- | --- | --- | --- | --- | --- | --- | --- | --- | --- | --- | --- | --- | --- |
| Performance indicators | | Forward passes (7-vs-7) | | Forward passes (11-vs-11) | | Offensive duels (7-vs-7) | | Offensive duels (11-vs-11) | | Defensive duels (7-vs-7) | | Defensive duels (11-vs-11) | |
|  | | Coeff. | SE | Coeff. | SE | Coeff. | SE | Coeff. | SE | Coeff. | SE | Coeff. | SE |
| Fixed Effects | Intercept | 0.78 | 0.10 | 0.54 | 0.12 | 0.66 | 0.15 | 0.61 | 0.15 | -0.21 | 0.14 | 0.05 | 0.18 |
|  | Team (U17) | 0.57 | 0.17 | -0.42 | 0.20 | 0.29 | 0.25 | -0.3 | 0.23 | -0.29 | 0.24 | 0.08 | 0.26 |
|  | Team (U19) | 0.54 | 0.15 | 0.14 | 0.20 | 0.61 | 0.24 | -0.16 | 0.24 | 0.07 | 0.21 | 0.65 | 0.29 |
|  | Team (U23) | 0.99 | 0.16 | 0.20 | 0.20 | 0.27 | 0.24 | -0.23 | 0.21 | 0.20 | 0.22 | 0.53 | 0.27 |
| Random Effect (SD) | Intercept | 0.22 | | 0.40 | | 0.35 | | 0.14 | | 0.37 | | 0.41 | |
| Coeff. = Estimated Regression Coefficient; SD = Standard Deviation; SE = Estimated Standard Error; The reference group for the factor  ‘Team’ is the Under 15 (U15) age category. | | | | | | | | | | | | | |
