# Supplementary material for: The validity of small-sided games in predicting 11-vs-11 soccer game performance
Source: PLoS One. 2020 Sep 21;15(9):e0239448. doi: 10.1371/journal.pone.0239448 (PMC7505454; doi:10.1371/journal.pone.0239448)
Supplement: S3 Table — (DOCX) [file pone.0239448.s003.docx]

| **S3 Table. Spearman’s correlations (95% CI in brackets) between the offensive performance indicators and shots on target (top), and defensive performance indicators and shots on target conceded (bottom), per age category and game format** | | | | | |
| --- | --- | --- | --- | --- | --- |
| **Team** | **Game format** | **Passes forward** | **Offensive duels** | **Chances created** | **Data points** |
| U15 | SSG | 0.04 (-0.26 – 0.34) | 0.26 (-0.05 – 0.52) | 0.57 (0.33 – 0.75) | 42 |
|  | 11-vs-11 | -0.26 (-0.89 – 0.70) | 0.32 (-0.66 – 0.90) | 0.40 (-0.61 – 0.92) | 6 |
| U17 | SSG | 0.41 (0.03 – 0.69) | 0.06 (-0.34 – 0.43) | 0.39 (0.01 – 0.68) | 26 |
|  | 11-vs-11 | 0.49 (-0.53 – 0.93) | -0.12 (-0.85 – 0.76) | 0.28 (-0.69 – 0.89) | 6 |
| U19 | SSG | 0.23 (-0.06 – 0.47) | 0.15 (0.13 – 0.42) | 0.39 (0.13 – 0.60) | 50 |
|  | 11-vs-11 | -0.06 (-0.83 – 0.79) | -0.26 (-0.89 – 0.70) | 0.78 (-0.09 – 0.97) | 6 |
| U23 | SSG | 0.26 (-0.04 – 0.51) | 0.20 (-0.10 – 0.47) | 0.57 (0.33 – 0.74) | 46 |
|  | 11-vs-11 | < .01 (-0.81 – 0.81) | 0.24 (-0.71 – 0.88) | 0.61 (-0.40 – 0.95) | 6 |

| **Team** | **Game format** | **Defensive duels** | **Pass interceptions** | **Applying pressure** | **Data points** |
| --- | --- | --- | --- | --- | --- |
| U15 | SSG | -0.15 (-0.43 – 0.16) | -0.01 (-0.31 – 0.30) | 0.04 (-0.26 – 0.34) | 42 |
|  | 11-vs-11 | 0.14 (-0.76 – 0.85) | 0.08 (-0.78 – 0.84) | 0.49 (-0.53 – 0.93) | 6 |
| U17 | SSG | -0.28 (-0.60 – 0.13) | -0.02 (-0.40 – 0.37) | -0.16 (-0.52 – 0.24) | 26 |
|  | 11-vs-11 | -0.6 (-0.95 – 0.42) | 0.67 (-0.32 – 0.96) | -0.48 (-0.93 – 0.54) | 6 |
| U19 | SSG | -0.06 (-0.33 – 0.22) | -0.07 (-0.35 – 0.21) | -0.14 (-0.40 – 0.14) | 50 |
|  | 11-vs-11 | -0.13 (-0.85 – 0.76) | -0.13 (-0.85 – 0.76) | -0.39 (-0.91 – 0.61) | 6 |
| U23 | SSG | -0.18 (-0.44 – 0.12) | -0.21 (-0.47 – 0.09) | -0.17 (-0.44 – 0.13) | 46 |
|  | 11-vs-11 | 0.31 (-0.67 – 0.90) | 0.12 (-0.76 – 0.85) | -0.19 (-0.87 – 0.74) | 6 |
