# Supplementary material for: The validity of small-sided games in predicting 11-vs-11 soccer game performance
Source: PLoS One. 2020 Sep 21;15(9):e0239448. doi: 10.1371/journal.pone.0239448 (PMC7505454; doi:10.1371/journal.pone.0239448)
Supplement: S4 Table — (DOCX) [file pone.0239448.s004.docx]

| **S4 Table. Mean (and SD) events per 6 minutes on the performance indicators across all age categories (top) and per age category (bottom).** | | | | |
| --- | --- | --- | --- | --- |
|  | SSG | | 11-vs-11 | |
| Performance indicator | Mean (SD) | 95% CI | Mean (SD) | 95% CI |
| Passes forward | 15.40 (3.71) | 14.83 - 15.97 | 13.35 (3.80) | 11.83 - 14.87 |
| Tackles | 4.62 (2.78) | 4.20 - 5.05 | 3.86 (1.50) | 3.26 - 4.46 |
| Take ons | 4.73 (2.76) | 4.30 - 5.15 | 3.73 (1.68) | 3.06 - 4.40 |
| Applying pressure | 3.20 (2.22) | 2.86 - 3.53 | 2.70 (1.53) | 2.09 - 3.31 |
| Pass interceptions | 2.52 (1.74) | 2.26 - 2.79 | 2.59 (1.13) | 2.13 - 3.04 |
| Defensive aerial duels | 0.24 (0.50) | 0.17 – 0.32 | 1.82 (1.12) | 1.31 – 2.28 |
| Staying in front | 2.37 (1.87) | 2.09 - 2.66 | 1.18 (0.68) | 0.90 - 1.45 |
| Offensive aerial duels | 0.29 (0.54) | 0.20 – 0.37 | 1.18 (0.82) | 0.85 – 1.51 |
| Dribbles | 1.43 (4.73) | 0.70 - 2.15 | 0.85 (3.73) | 0* - 2.34 |
| Shots on target | 1.35 (1.19) | 1.17 - 1.54 | 0.42 (0.35) | 0.28 - 0.56 |
| Shots | 2.20 (1.48) | 1.97 - 2.43 | 0.80 (0.48) | 0.60 - 0.99 |
| Chances created | 0.72 (0.89) | 0.58 - 0.86 | 0.27 (0.19) | 0.19 - 0.34 |

| **S4 Table - *continued*** | | | | | | | | | | | | |
| --- | --- | --- | --- | --- | --- | --- | --- | --- | --- | --- | --- | --- |
| **Team** | **Game format** | **Passes forward** | **Tackles** | **Dribbles** | **Take ons** | **Staying in front** | **Offensive aerial duels** | **Defensive aerial duels** | **Applying pressure** | **Pass interceptions** | **Shots on target** | **Chances created** |
| 15 | SSG | 14.33 (3.84) | 6.19 (3.00) | 1.88 (1.67) | 5.76 (3.16) | 2.07 (1.94) | 0.21 (0.47) | 0.19 (0.40) | 2.95 (2.27) | 3.02 (1.99) | 1.17 (1.31) | 0.79 (1.00) |
|  | 11-vs-11 | 18.34 (1.97) | 5.34 (0.75) | 1.60 (1.20) | 5.69 (1.48) | 1.00 (0.40) | 1.26 (0.76) | 1.80 (1.29) | 3.51 (1.77) | 3.31 (0.97) | 0.66 (0.49) | 0.37 (0.20) |
| 17 | SSG | 15.65 (4.19) | 4.54 (1.96) | 1.50 (1.39) | 4.96 (2.34) | 2.39 (1.58) | 0.54 (0.76) | 0.35 (0.56) | 3.69 (2.60) | 2.39 (1.55) | 1.42 (1.30) | 0.77 (0.86) |
|  | 11-vs-11 | 10.55 (3.13) | 4.55 (1.10) | 1.15 (0.84) | 2.75 (1.32) | 1.43 (1.21) | 1.00 (0.49) | 1.75 (0.71) | 2.45 (1.97) | 2.88 (0.62) | 0.28 (0.28) | 0.30 (0.25) |
| 19 | SSG | 15.76 (3.49) | 4.72 (2.78) | 1.60 (1.47) | 4.60 (2.67) | 2.58 (2.19) | 0.30 (0.51) | 0.20 (0.50) | 2.84 (2.02) | 2.82 (1.84) | 1.42 (1.21) | 0.54 (0.73) |
|  | 11-vs-11 | 13.42 (0.99) | 2.47 (1.17) | 0.78 (0.54) | 3.69 (1.39) | 0.91 (0.50) | 1.29 (1.09) | 2.22 (1.47) | 2.91 (1.25) | 2.04 (1.41) | 0.42 (0.26) | 0.22 (0.14) |
| 23 | SSG | 15.85 (3.46) | 3.13 (2.17) | 1.07 (1.02) | 3.78 (2.40) | 2.41 (1.59) | 0.20 (0.45) | 0.28 (0.54) | 3.52 (2.11) | 1.83 (1.22) | 1.41 (1.00) | 0.83 (0.95) |
|  | 11-vs-11 | 11.09 (2.53) | 3.09 (0.99) | 0.62 (0.44) | 2.80 (0.61) | 1.38 (0.18) | 1.18 (1.00) | 1.56 (1.08) | 1.93 (0.71) | 2.11 (1.10) | 0.33 (0.28) | 0.18 (0.14) |
