# Supplementary material for: The validity of small-sided games in predicting 11-vs-11 soccer game performance
Source: PLoS One. 2020 Sep 21;15(9):e0239448. doi: 10.1371/journal.pone.0239448 (PMC7505454; doi:10.1371/journal.pone.0239448)
Supplement: S5 Table — (DOCX) [file pone.0239448.s005.docx]

| **S5 Table. Spearman’s correlations (95% CI in brackets) between the performance indicators in the SSGs and 11-vs-11 games, per age category (i.e. team)** | | | | | | | | | |
| --- | --- | --- | --- | --- | --- | --- | --- | --- | --- |
| **Team** | **Passes forward** | **Chances created** | **Shots on target** | **Pass Interceptions** | **Pressure** | **Offensive duels** | **Overall offensive performance** | **Defensive duels** | **Overall defensive performance** |
| U15 | 0.08 (-0.42 – 0.54) | -0.1 (-0.56 - 0.40) | 0.69 (0.32 – 0.88) | 0.71 (0.34 – 0.89) | 0.18 (-0.33 – 0.61) | 0.49 (-0.01 – 0.79) | 0.53 (0.04 – 0.81) | 0.08 (-0.42 – 0.54) | 0.20 (-0.31 – 0.62) |
| U17 | 0.33 (-0.22 – 0.72) | 0.18 (-0.36 - 0.64) | 0.13 (-0.41 – 0.60) | 0.32 (-0.23 – 0.71) | 0.47 (-0.06 – 0.79) | -0.08 (-0.59 – 0.47) | 0.49 (-0.05 – 0.81) | 0.13 (-0.41 – 0.60) | 0.19 (-0.36 – 0.64) |
| U19 | 0.40 (-0.12 – 0.75) | -0.11 (-0.58 - 0.41) | 0.43 (-0.08 - 0.76) | 0.69 (0.30 – 0.89) | 0.22 (-0.31 – 0.65) | 0.38 (-0.19 – 0.76) | 0.53 (0 – 0.83) | -0.02 (-0.53 – 0.49) | 0.27 (-0.28 – 0.69) |
| U23 | 0.65 (0.21 – 0.87) | 0.04 (-0.48 – 0.54) | 0.09 (-0.44 – 0.58) | 0.24 (-0.31 – 0.67) | 0.68 (0.25 – 0.88) | 0.51 (0 – 0.81) | 0.24 (-0.31 – 0.67) | -0.10 (-0.60 – 0.46) | 0.45 (-0.11 – 0.79) |
