# Supplementary material for: The validity of small-sided games in predicting 11-vs-11 soccer game performance
Source: PLoS One. 2020 Sep 21;15(9):e0239448. doi: 10.1371/journal.pone.0239448 (PMC7505454; doi:10.1371/journal.pone.0239448)
Supplement: S6 Table — (DOCX) [file pone.0239448.s006.docx]

| **S6 Table. Spearman’s correlations (95% CI in brackets) between physiological and motor tests and overall offensive (top) and defensive performance (bottom) in 11-vs-11 games, per age category (i.e. team)** | | | | |
| --- | --- | --- | --- | --- |
| **Team** | **10 m sprint** | **30 m sprint** | **ISRT** | **Agility** |
| U15 | -0.07 (-0.62 – 0.52) | 0.02 (-0.56 – 0.59) | -0.26 (-0.68 – 0.29) | 0.08 (-0.51 – 0.63) |
| U17 | -0.58 (-0.85 – -0.06) | -0.66 (-0.88 – -0.20) | 0.53 (-0.01 – 0.83) | -0.55 (-0.84 – -0.03) |
| U19 | 0.04 (-0.50 – 0.56) | 0.09 (-0.46 – 0.59 | 0.33 (-0.24 – 0.73) | 0.31 (-0.27 – 0.72) |
| U23 | -0.07 (-0.56 – 0.46) | -0.1 (-0.58 – 0.43) | -0.03 (-0.53 – 0.49) | -0.17 (-0.63 – 0.37) |
| U15 | 0.04 (-0.55 – 0.60) | -0.11 (-0.64 – 0.50) | -0.07 (-0.55 – 0.44) | 0.20 (-0.42 – 0.70) |
| U17 | 0.10 (-0.43 – 0.58) | 0.10 (-0.43 – 0.58) | -0.30 (-0.70 – 0.25) | 0.22 (-0.33 – 0.65) |
| U19 | 0.02 (-0.49 – 0.53) | -0.11 (-0.59 – 0.43) | 0.01 (-0.51 – 0.52) | -0.24 (-0.67 – 0.31) |
| U23 | 0.05 (-0.49 – 0.57) | 0.19 (-0.38 – 0.66) | -0.09 (-0.59 – 0.46) | 0.31 (-0.26 – 0.72) |
