# Supplementary material for: The validity of small-sided games in predicting 11-vs-11 soccer game performance
Source: PLoS One. 2020 Sep 21;15(9):e0239448. doi: 10.1371/journal.pone.0239448 (PMC7505454; doi:10.1371/journal.pone.0239448)
Supplement: Attachment — Submitted filename: Summary of revisions.docx [file pone.0239448.s008.docx]

Reviewer #1

General comments:

*Thank you for submitting your revised manuscript to PLoS One. The study still presents an issue that is relevant to the field of talent research in soccer. From my point of view, the authors provide an extensive and accomplished revision of their manuscript for which they should be complimented. They addressed most of the reviewers’ comments in an adequate and satisfying way. Consequently, I would recommend the paper to be published, after some further smaller concerns have been addressed.*

**REPLY:** Thank you for your positive evaluation of the manuscript. We are glad to hear that you still consider the manuscript relevant, and we believe that the quality of the manuscript has further improved. Please see our reply to your suggestions below.

Specific comments:

1) Abstract, l23.ff: Please revise the first sentence as “performance” is too general and “in soccer talent” seems a bit confusing. Suggestion: Predicting game performance has been a major focus within talent identification and development/talent promotion”

**REPLY:** Agreed, we have changed this sentence to “Predicting performance in soccer games has been a major focus within talent identification and development” (see p.2 L.24-25)

2) Abstract, l.33: specify predictive validity here: “predictive validity for 11-vs-11 game performance”.

**REPLY:** This has been addressed (see p.2, L.33-34)

3) Abstract, l.41: remove “ability”

**REPLY:** This has been addressed (see p.2, L.42)

4) L.50 and L. 437: remove hyphen before “identification”/” development”

**REPLY:** This has been addressed (see p.3 L.50 and p.22, L.443)

5) L.69: provide an example for these inconsistencies regarding performance levels.

**REPLY:** We have added the following example from the study by Swann et al. (2016): “For example, definitions of elite athletes have ranged from international to regional level competitors, and strongly depend on the competitiveness of the sport in the athlete’s country” (see p.3-4, L.69-71)

6) L.81-84: provide reference

**REPLY:** this has been addressed (see p. 4, L.86)

7) L.134: change the order to “concurrent or predictive” in line with the enumeration of the cited studies.

**REPLY:** this has been addressed (see p.6, L.138)

8) L.138: present r’s in italics.

**REPLY:** this has been addressed (see p.6, L.142)

9) L.164: Think about removing “the oldest” as this could cause confusion. Insert “… from under-15 years (U15), U17, U19, and U23 teams of a …” instead.

REPLY: this has been addressed. Since we already specify in L.135 that U13 stands for under-13 year old players, we have shortened it to U15 here (see P.8, L.169-170)

10) L.172: replace “away from” the average by “less than”?

**REPLY:** this has been addressed; we have changed ‘away from’ to ‘below’ (see p.8, L.176)

11) L.174: remove “under-15 years here” (see comment 9).

**REPLY:** this has been addressed (see p.8, L.178)

12) Table 1: Title: “in brackets” instead of “between parentheses”?

**REPLY:** this has been addressed (see p.8, Table 1)

13) Table 1: please provide sample sizes here as well.

**REPLY:** this has been addressed (see p.8, Table 1)

14) L.270: Although it seems to be clear, please refer to the part “notational analyses” or write directly that you assessed the criterion also by performing notational analysis.

**REPLY:** this has been addressed; we have emphasized that we used the same notational analysis procedure for analyzing the 11-vs-11 games as for the SSGs (see p.12, L.273-274)

15) L.326: remove “on”?

**REPLY:** this has been addressed (see p.16, L.331)

16) L.343 ff.: provide a reference.

**REPLY:** We have added the following reference here: Borenstein et al. (2010). A basic introduction to fixed-effect and random-effects models for meta-analysis. Research Synthesis Methods, 1(2), 97–111. (see p.17, L.350)

17) L.372: I wonder why you did not perform an additional Chi-square GoF analysis as well (after removing aerial duels. This would be consistent and would strengthen the thread of this part.

**REPLY:** Agreed, we have performed an additional Chi-square goodness of fit test, and report its results in the revised manuscript (see p.18, L.378-379)

18) L.477ff/L.490ff: I am not finally convinced with the reaction on the comments of R1 and R2 regarding the reasons why correlations of the isolated physiological and motor tests with 11vs11 game performance were quite small. I am convinced that these small values are mainly since 11vs11 performance was assessed solely by on-ball-parameters. An integration of physiological and tactical (off-ball) would likely would have led to higher correlations. I appreciate, that the authors already elaborated on this, but would prefer that this fact is more highlighted and discussed. In my point of view, this could happen within the paragraph L.477ff or within L.490ff and would further strengthen the manuscript.

**REPLY:** We have highlighted this issue more clearly in the revised manuscript, and added that: “Integrating such (physiological) measures into our on-ball 11-vs-11 performance metrics could have increased the predictive validities of the physiological and motor tests [59].” (see p.25, L.508 - 510).

19) L.504 ff: Think about including a short part here that discusses your way of assessing the 11vs11 performance indicators (i.e., notational analyses). This proceeding is in parts a subjective one. Maybe other techniques (as mentioned in the introduction) could provide additional objective information that could improve the assessment of the criterion. This could be a perspective for future researchers.

**REPLY:** Agreed, we elaborate on the limitations of notational analysis in the revised manuscript: “Other limitations pertain to the notational analysis method used to assess soccer performance. This is a relatively intensive method to assess performance and its reliability depends on a common interpretation of indicators by each coder. Although the reliability was acceptable in our study, it is almost unavoidable that particular definitions of indicators (e.g., ‘applying pressure’) leave room for interpretation. Additionally, using the same observers to code both the predictor and criterion data could have positively affected the correlations between the indicators. Integrating physiological or tactical information derived through local or global positioning systems into the predictor or criterion may offer more reliable information. This could improve soccer performance assessments, and future research should consider if this is feasible.” (see p.25 – 26, L.512 – 521)

Reviewer #2:

It seems to me that the authors invested a lot of time and thoughts to revise the manuscript extensively based on the reviewers’ comments. Therefore, I would recommend the paper for publishing in PLOS ONE. There are only a few comments that should be considered before publishing the manuscript.

**REPLY:** Thank you for your positive evaluation of the manuscript. We are glad to hear that you would recommend the paper for publication. Please see our reply to your comments below.

SPECIFIC COMMENTS

• p.3., l. 54 and ff.: use *sprint* instead of *speed*

**REPLY:** Given that we would like to refer to the attribute here, rather than solely the test, we have changed ‘speed’ to ‘sprint speed’ in the revised manuscript (see p.3, L.54; p.5, L.103 & L.105)

• p.4., l. 103: insert spaces between > and 20m

**REPLY:** this has been addressed (see p.5, L.103 & L.104)

• p.4., l. 113: what is about *motor skills*?

**REPLY:** this has been addressed; we have added motor skills here (see p.5, L. 114)

• p.5., l. 125: use the same row of the enumeration such as on p. 4., l. 113.

**REPLY:** this has been addressed (see p.6, L.127 -128)

• p.5., l. 133: could you provide age groups investigated in this study by Olthof et al.? such as on p. 4., l. 113.

**REPLY:** this has been addressed; we have added the age groups here (see p.6, L.135 – 136)

• p.8., Table 1: Thank you for providing this table. Please indicate the number of players per age group.

**REPLY:** this has been addressed (see p.8 Table 1)

• p.15., l. 326: delete *on*

**REPLY:** this has been addressed (see p.16, L.331)

• p.16., l. 334: name the specific test in brackets.

**REPLY:** this has been addressed (see p. 17, L.339)
